# In Situ Vaccination with Mitochondria‐Targeting Immunogenic Death Inducer Elicits CD8^+^ T Cell‐Dependent Antitumor Immunity to Boost Tumor Immunotherapy

**DOI:** 10.1002/advs.202300286

**Published:** 2023-05-01

**Authors:** Yuxiang Wang, Weiran Wang, Rong Gu, Jing Chen, Qian Chen, Tingsheng Lin, Jinhui Wu, Yiqiao Hu, Ahu Yuan

**Affiliations:** ^1^ State Key Laboratory of Pharmaceutical Biotechnology Medical School and School of Life Science Nanjing University Nanjing 210093 P. R. China; ^2^ Department of Urology Nanjing Drum Tower Hospital The Affiliated Hospital of Nanjing University Medical School Nanjing 210093 P. R. China; ^3^ Jiangsu Key Laboratory for Nano Technology Nanjing University Nanjing 210093 P. R. China

**Keywords:** calreticulin, immunogenic cell death, in situ tumor vaccination, mitochondria‐targeting, type I interferon

## Abstract

In situ vaccination can elicit systemic antitumor immunity to potentiate immune checkpoint blockade (ICB) in poorly immunogenic tumors. Herein, an immunogenic cell death (ICD) inducer for in situ vaccination, which is based on a mitochondria‐targeting modification of fenofibric acid (FFa), a lipid‐lowering drug with potential inhibitory efficacy of respiratory complex I is developed. Mitochondria‐targeting FFa (Mito‐FFa) inhibits complex I efficiently and increases mitochondrial ROS (mtROS) generation, which further triggers endoplasmic reticulum (ER) stress with unprecedented calreticulin (CRT) exposure on tumor cellular membranes. Moreover, the generated mtROS also oxidizes mitochondrial DNA (mtDNA) and promotes it leakage into the cytoplasm for cGAS‐STING‐dependent type I interferon (IFN‐I) secretion. The synchronous CRT exposure and IFN‐I secretion successively improve the uptake of tumor antigens, maturation of dendritic cells (DCs) and cross‐priming of CD8^+^ T cells. In a poorly immunogenic 4T1 tumor model, a single intratumoral (i.t.) Mito‐FFa injection turns immune‐cold tumors into hot ones and elicits systemic tumor‐specific CD8^+^ T cells responses against primary and metastatic tumors. Furthermore, the synergistic effect with PD‐L1 blockade and good bio‐safety of i.t. Mito‐FFa administration suggest the great translational potential of Mito‐FFa in tumor immunotherapy.

## Graphical abstract

The mitochondria‐targeting immunogenic death inducer Mito‐FFa, modified based on fenofibric acid, is developed to amplify mtROS level of tumor cells for in situ tumor vaccination. Results show that a single intratumoral Mito‐FFa injection obviously induces CD8^+^ T cell‐mediated systemic antitumor immunity by coinducing calreticulin exposure and IFN‐I secretion and synergizes with anti‐PD‐L1 to control primary and metastatic tumors.

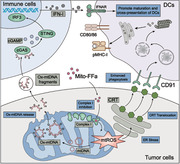

## 1 Introduction

Although immune checkpoint blockade (ICB) has revolutionized tumor therapies, novel combinatorial strategies are still required to improve its response in poorly immunogenic tumors.^[^ ^1^ ^]^ Herein, an approach gaining interest in the immuno‐oncology community is in situ tumor vaccination. In which, primary tumors upon proper treatments would serve as a depot of tumor antigens and adjuvant, and in situ mobilize systemic immune responses against distant lesions.^[^ ^2^ ^]^ For effective in situ vaccination, several immune events should be coordinated coherently, including the exposure and uptake of tumor antigens, priming of tumour‐special CD8^+^ T cell responses and the forming of immunoactive tumor microenvironment (TME).^[^ ^2b^ ^]^ Antigen presenting cells (APCs) phagocytizing dying tumor cells is the initial stage of antitumor immunity activation.^[^ ^3^ ^]^ Calreticulin (CRT) exposed on tumor cell membrane during immunogenic cell death can act as an “eat me” signal for APCs and mediate more efficient antigen uptake.^[^ ^4^ ^]^ Subsequently, CD8^+^ T cells should be primed to exert tumor cytotoxic effects. IFN‐I is indispensable to cross‐presentation of exogenous antigens, which is a crucial mechanism for generating CD8^+^ T cell dependent responses.^[^ ^5^ ^]^ It can induce indigestion of exogenous antigens by maintaining a higher pH of APCs phagosomes to allow antigens to escape into the cytosol, where they are degraded by the proteasome and then cross‐presented.^[^ ^6^ ^]^ Moreover, IFN‐I can also support the functions of immune cells and relieve immunosuppressive TME for optimal antitumor immunity.^[^ ^7^ ^]^ Thus, synchronous CRT exposure and IFN‐I secretion are crucial for the success of in situ vaccination.

Due to the presence of ER‐mitochondria interface, mtROS can diffuse to ER and potentially cause ER stress,^[^ ^8^ ^]^ which would further trigger a series of unfolded protein responses (UPR), including CRT exposure.^[^ ^9^ ^]^ Meanwhile, mitochondrial control of immunity is currently of intense interest. Mitochondria containing various damage associated molecular patterns (DAMPs).^[^ ^10^ ^]^ For instance, cytochrome C, heme, and mtDNA, are classic inflammatory factors to stimulate many pattern recognition receptors (PRRs).^[^ ^11^ ^]^ Impressively, studies demonstrated that mtDNA could escape from stressed mitochondria (e.g., oxidative damage) into cytoplasm and potentially activate cGAS‐STING pathway for IFN‐I secretion.^[^ ^12^ ^]^ Therefore, mtROS might be a viable target to synergistically promote CRT exposure and IFN‐I secretion. MtROS is mainly contributed by the electron (e^−^) leakage from the mitochondrial electron transport chain^[^ ^13^ ^]^ and many studies showed inhibition of respiratory complex I or complex III could potentiate e^−^ leakage and generate abundant mtROS.^[^ ^14^ ^]^ Fenofibrate (FF) is an FDA‐approved lipid‐lowering drug relying on its metabolite, fenofibric acid (FFa), to activate PPAR*α*. Some recent in vitro studies reported that unhydrolyzed FF could inhibit complex I and enhance mtROS generation,^[^ ^15^ ^]^ showing the potential as an ICD inducer. However, this efficacy is weak in vivo. Most of FF would be inevitably degraded into FFa by esterases, and the hydrophilic carboxyl group in FFa prevented its accumulation in mitochondria.^[^ ^16^ ^]^ We, therefore, hypothesized that metabolic properties of FF/FFa may limit their abilities in producing mtROS, which could reduce their efficacy in tumor immunotherapy.

**Figure 1:**
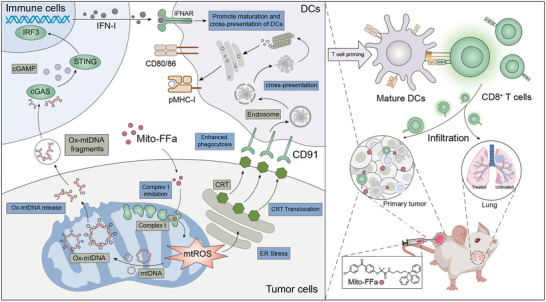
Mito‐FFa induces in situ tumor vaccination against primary and metastatic tumors.

Therefore, we synthesized Mito‐FFa by conjugating triphenylphosphonium (TPP) to FFa to endow it with mitochondrial‐targeting capacity. Our results showed that Mito‐FFa inhibited respiratory complex I and increased mtROS level efficiently, exhibiting the tumor‐cell‐specific toxicity and nearly 10–20 folds more potent cytotoxicity than FF. More importantly, Mito‐FFa possessed surprising ability in inducing CRT exposure. At its IC_50_, Mito‐FFa induced 84.9% living tumor cells to expose CRT and mediated potent phagocytosis of APCs, which was far stronger than previously reported ICD inducers.^[^ ^17^ ^]^ Meanwhile, Mito‐FFa treatment also resulted in obvious mtDNA leakage, and then activated the cGAS/STING signaling pathway for IFN‐I secretion from surrounding immune cells. In the poorly immunogenic 4T1 breast tumor model, only a single intratumoral Mito‐FFa injection was able to greatly enhance the immunogenicity of tumors, relieving the immunosuppressive TME and induce systemic antitumor immunity against primary and metastatic tumors. Data on Batf3^−/−^ mice and antibody‐mediated depletion indicated vaccine effect of Mito‐FFa was depended on cross‐priming of CD8^+^ T cells. Notably, Mito‐FFa treatment effectively sensitized tumors to anti‐PD‐L1 with 57% (4/7) complete tumor regression ratio and lightest lung metastasis. Thus, Mito‐FFa would be a powerful immunostimulatory agent severing as an outstanding partner to established ICB therapies (**Figure** **1**).

## 2 Results

### 2.1 Mito‐FFa Inhibited Tumor Cells Growth Via Interfering with Mitochondrial Function

**Figure 2:**
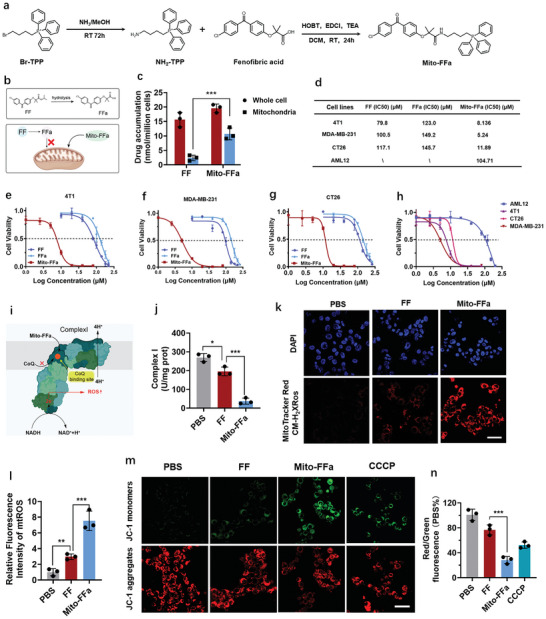
Mito‐FFa disturbed mitochondrial function and impeded tumor cell growth. a) Synthesis of Mito‐FFa. b,c) Different accumulation of Mito‐FFa within mitochondria or whole tumor cells. Data are shown as mean ± SD (*n* = 3, one‐way ANOVA). d) The IC_50_ for each cell line was calculated using PRISM. e–h) The dose‐cytotoxicity curves of FF, FFa, and Mito‐FFa in various cell lines. Data are shown as mean ± SD (*n* = 3). i) Illustration of Mito‐FFa‐induced complex I inhibition. j) The inhibitory effects of FF and Mito‐FFa against mitochondrial respiratory complexes I. Data are shown as mean ± SD (*n* = 3, one‐way ANOVA). k,l) The fluorescence images k) and quantification l) of mtROS induced by FF and Mito‐FFa. Scale bar = 50 µm. Data are shown as mean ± SD (*n* = 3, one‐way ANOVA). m) The fluorescent images of 4T1 cells stained by JC‐1. Scale bar = 50 µm. n) Quantification of red to green JC‐1 fluorescence intensity normalized to control. Data are shown as mean ± SD (*n* = 3, one‐way ANOVA). N.S. represents nonsignificance, and *p < 0.05; **p < 0.01; ***p < 0.001.

Mito‐FFa was synthesized according to the reaction depicted in **Figure** **2**a and characterized by mass spectrometry (Figure S1, Supporting Information), nuclear magnetic resonance (Figures S2 and S3, Supporting Information), and X‐ray photoelectron spectroscopy (Figure S4, Supporting Information), respectively. To determine whether Mito‐FFa could localize within mitochondria more efficiently, we detected the content of Mito‐FFa in whole cells or isolated mitochondria after 12‐h coincubation. Compared with FF, the uptake of Mito‐FFa in cells and mitochondria were increased 1.3 and 4.5‐fold, respectively (Figure 2b,c). Next, we evaluated the cytotoxicity of FF, FFa, and Mito‐FFa in a variety of cell lines. FFa had comparable cytotoxicity to FF. But when compared with FF, Mito‐FFa exerted 9.8, 19.2, and 9.8‐fold lower IC_50_ values on breast 4T1 tumor cells, breast MDA‐MB‐231 tumor cells and colon CT26 tumor cells, respectively. It could be ascribed to TPP‐mediated mitochondria targeting (Figure 2d–g). Then, normal alpha mouse liver 12 (AML12) cells, a type of liver parenchymal cell, were utilized to detect whether the cytotoxicity of Mito‐FFa was tumor cell‐specific. The IC_50_ of Mito‐FFa to AML12 cells was detected at 104.7 µm, which was much higher than all three tumor cell lines (Figure 2d,h). The significant difference of cytotoxicity between tumor cells and AML12 cells was probably derived from the hyper‐polarized mitochondrial membrane potential (MMP) of tumor cells, which facilitated the selective accumulation of TPP conjugates within mitochondria.^[^ ^18^ ^]^

Next, the effects of Mito‐FFa and FF on mitochondrial structure and function were examined on 4T1 tumor cells. First, we found that the inhibition of Mito‐FFa to respiratory complex I (inhibition rate 86.1%) was much more potent than FF (27.1%, Figure 2i,j) at the same concentration (8 µm), which should be related to more effective mitochondrial accumulation of Mito‐FFa. Respiratory complex I is a metabolic enzyme coupling electron transfer from NADH to ubiquinone for translocation of protons across the membranes. It was widely reported that the inhibition of complex I would cause more electron leak, and then reacted with O_2_ to produce more mtROS. Thus, we next evaluated the level of mtROS by Mitotracker Red CM‐H2XRos (a probe that is located within mitochondria and remains nonfluorescent until it is oxidized by ROS) upon the treatments of Mito‐FFa and FF at their respective IC_50_ concentrations. As shown in Figure 2k,l, only weak mtROS red fluorescence within FF (80 µm)‐treated 4T1 breast tumor cells could be detected. It showed that the major antitumor target of FF is not mitochondria, which is consistent with previous studies. However, Mito‐FFa (8 µm) induced significantly brighter red fluorescence, indicating high‐level mtROS generation. To further clarify the species of mtROS induced by Mito‐FFa, a MitoSOX‐based flow cytometric assay was used. The results shown in Figure S5 (Supporting Information)confirmed the mitochondrial superoxide anion generation. Excessive mtROS could potentially lead to mitochondria depolarization. In Figure 2m,n, JC‐1 staining showed that Mito‐FFa (8 µm)‐treated cells exhibited more serious Δ*ψ*m loss, when compared with FF (80 µm) or CCCP (positive control). Overall, all these results demonstrated that the excessive generation of mtROS and superior antitumor effects would be obtained by targeting fenofibric acid to mitochondria.

### 2.2 Mito‐FFa Induced ER Stress for CRT Exposure and Enhanced Phagocytosis

**Figure 3:**
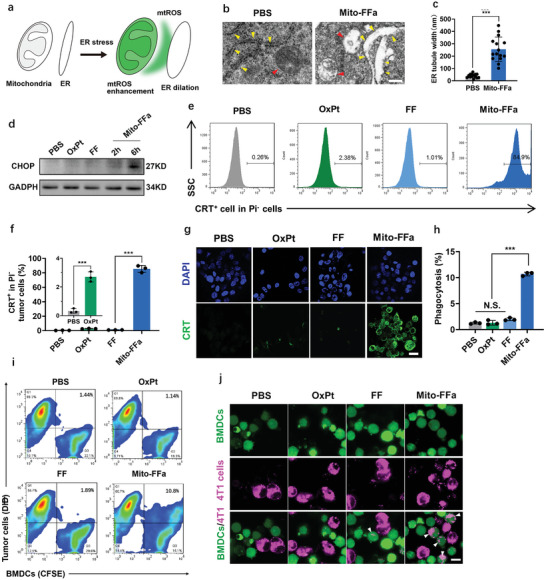
ER stress induced by Mito‐FFa improved CRT exposure and phagocytosis. a) Illustration of mtROS‐induced ER stress. b) TEM of 4T1 tumor cells treated by PBS (phosphate buffered saline) and Mito‐FFa. ER and mitochondria denoted by yellow and red arrowheads, respectively. c) Histogram comparing distribution of ER tubule width between untreated and treated samples based on b). Data are shown as mean ± SD (*n* = 15, unpaired two‐tailed Student's *t*‐test). d) Western blot of CHOP and GADPH. e,f) Representative flow cytometry images e) and quantification f) of PI^−^/CRT^+^ 4T1 tumor cells. Data are shown as mean ± SD (*n* = 3, one‐way ANOVA). g) Immunofluorescence of 4T1 cells stained with anti‐CRT antibody. Scale bar = 20 µm. h,i) Representative flow cytometry plots and phagocytosis (%) of BMDCs. Data are shown as mean ± SD (*n* = 3, one‐way ANOVA). j) The phagocytosis of 4T1 tumor cells by BMDCs observed under CLSM. Scale bar = 10 µm. N.S. representing nonsignificance, and *p < 0.05; **p < 0.01; ***p < 0.001.

Some mitochondria and ER are physically connected to form mitochondria associated ER membranes (MAMs), which participate in fundamental biological processes. For instance, M. Booth et.al., reported that mtROS could be diffused to ER via MAMs, thereby inducing ER oxidative stress^[^ ^19^ ^]^ (**Figure** **3**a). Therefore, we investigated whether Mito‐FFa‐mediated mtROS would cause ER stress and subsequent CRT exposure. Theoretically, ER stress is often accompanied by morphological changes.^[^ ^20^ ^]^ As shown in Figure 3b,c, transmission electron microscope (TEM) showed that obvious ER dilation and loss of contents were substantially occurred in Mito‐FFa‐treated 4T1 cells but not in the control group. Meanwhile, the up‐expression of CHOP (Figure 3d) and p‐eIF2*α* (Figure S6, Supporting Information) upon the treatment of Mito‐FFa indicated the initiation of the unfolded protein response (UPR). These data confirmed Mito‐FFa treatment could induce ER stress within tumor cells.

We then compared the capacity of Mito‐FFa, FF, and Oxaliplatin (OxPt, a well‐established classic ICD inducer) in inducing CRT exposure at their respective IC_50_ concentrations. Flow cytometry data in Figure 3e,f showed OxPt (40 µm) induced CRT exposure as expected. However, we surprisingly observed that upon incubation of Mito‐FFa (8 µm), 84.9% of living tumor cells (Pyridine iodide negative) exposed CRT, while exposure occurred in only 1% of cells treated with FF. Meanwhile, Mito‐FFa‐induced CRT exposure showed a 35.7‐fold enhancement to OxPt treatment. Similar results were also demonstrated by confocal laser scanning microscopy (CLSM) (Figure 3g; and Figure S7, Supporting Information). Surface‐exposed CRT is an “eat me” signal for APCs and promotes the phagocytosis of cancer cells through the binding with CD91 on APCs. Thus, we evaluated the phagocytosis promoting effect of Mito‐FFa via flow cytometry and CLSM. First, Raw264.7 (monocyte/macrophage like cells) were cocultured with 4T1 cells that pretreated with FF, OxPt, and Mito‐FFa, respectively for 12 h. As shown in Figure S8 (Supporting Information), the phagocytic percentages of Mito‐FFa‐treated cells were substantially enhanced to 32.5%, which was significantly higher than those of untreated tumor cells (12.3%), FF‐treated (14.4%), and OxPt‐treated cells (13.9%). To reduce nonspecific phagocytosis, bone‐marrow derived dendritic cells (BMDCs) were also introduced for comparison. As shown in Figure 3h,i, Mito‐FFa‐treated cells were also engulfed most effectively by BMDCs. To further confirm the phagocytosis of 4T1 tumor cells by BMDCs, we observed the cellular phagocytosis with CLSM. In agreement with the results from flow cytometry, Mito‐FFa treatment led to a substantial increase of pink fluorescent tumor cells engulfed by BMDCs (Figure 3j). Therefore, these results suggested that high level of CRT exposure induced by Mito‐FFa would obviously promote their phagocytosis, which potentially ensured the sufficient uptake of tumor antigens.

### 2.3 Mito‐FFa Induced Ox‐mtDNA Leakage to Promote cGAS‐STING‐Dependent IFN‐I Secretion

**Figure 4:**
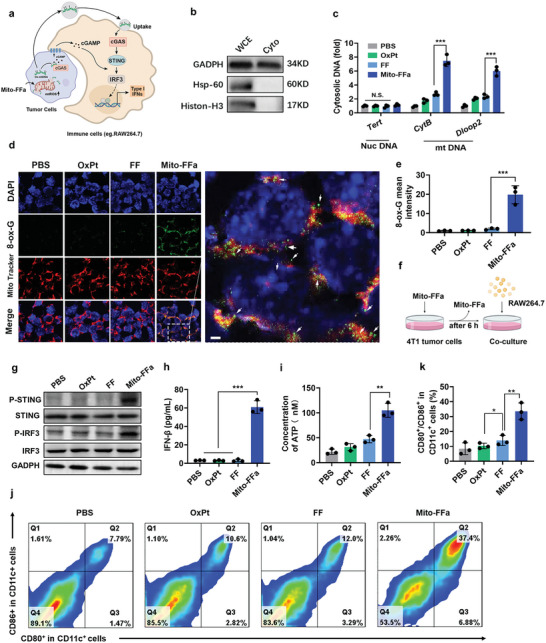
Mito‐FFa induced Ox‐mtDNA leakage for IFN‐I secretion. a) Illustration of Mito‐FFa‐mediated IFN‐I secretion by surrounding immune cells. b) Western blot verification of our cytosol fractionation protocol. Whole‐cell extracts (WCE) and cytosolic extracts (Cyto) were blotted using the indicated antibodies. c) The qPCR quantification of DNA in cytosolic extracts from treated 4T1 tumor cells. Data are shown as mean ± SD (*n* = 3, one‐way ANOVA). d) Representative images of MitoTracker Red CMXRos (red), 8‐ox‐G immunostaining (green), and DAPI (blue) in 4T1 cells after various treatments. The orange areas in the merged images indicated that 8‐ox‐G was colocalized with mitochondria, whereas the free green foci represented ox‐mtDNA leakage. Scale bar = 5 µm. e) The quantification of fluorescent intensity of 8‐ox‐G based on d). f) Illustration of the coculture system of Raw264.7 and Mito‐FFa‐treated cells. g) Western blot of p‐STING, STING, p‐IRF3, IRF3, and GADPH. h) Detection of IFN‐*β* levels in the Raw264.7/4T1 tumor cells coculture media by ELISA. Data are shown as mean ± SD (*n* = 3, one‐way ANOVA). i) Detection of ATP secretion. Data are shown as mean ± SD (*n* = 3, one‐way ANOVA). j,k) Representative flow cytometry images j) and quantification k) of mature DCs. Data are shown as mean ± SD (*n* = 3, one‐way ANOVA). N.S. represents nonsignificance, and *p < 0.05; **p < 0.01; ***p < 0.001.

Excessive mtROS would potentially increase mitochondrial membrane permeability and induce cytoplasmic release of mtDNA to activate the cGAS‐STING signal pathway for IFN‐I secretion (**Figure** **4**a). To test this hypothesis, we tried to fractionate 4T1 cells and then purified DNA from cytosolic extracts for qPCR analysis. Western blot analysis verified the effectiveness of our cytosol fractionation protocol (Histone‐H3, Hsp‐60, and GAPDH were used to determine nuclear, mitochondrial, and cytosolic fractions, respectively) (Figure 4b). The qPCR analysis indicated that more mtDNA (as represented by Dloop2 and CytB) instead of nuclear DNA (as represented by Tert) was enriched within the cytoplasm of Mito‐FFa‐treated 4T1 tumor cells (Figure 4c), confirming the mtDNA leakage mediated by Mito‐FFa treatment.

It was reported that mtDNA was more easily attacked by ROS due to the lack of adequate DNA repair mechanisms.^[^ ^21^ ^]^ Moreover, oxidized mtDNA (Ox‐mtDNA) was widely reported to be resistant to DNase degradation and could activate the cGAS‐STING signal pathway more efficiently.^[^ ^22^ ^]^ Thus, we next evaluated the oxidized state of mtDNA upon different treatments by detecting the level of 8‐oxo‐Gua (8‐Oxoguanine), a hallmark of oxidative DNA damage. As shown in Figure 4d,e, Mito‐FFa‐treated 4T1 cells exhibited significant increased green fluorescence of anti‐8‐oxo‐G antibody, but no obvious fluorescence was observed in other groups. In addition, the mainly colocalization of green fluorescence with MitoTracker Red CMXRos also confirmed the specific disruption of mitochondria by Mito‐FFa. However, some fluorescent foci of anti‐8‐oxo‐G antibody also appearing outside the mitochondria, might indicating the leakage of Ox‐mtDNA.

We then investigated whether these Ox‐mtDNA escaping from stressed mitochondria would activate the cGAS‐STING pathway. Raw264.7, when cocultured with Mito‐FFa‐treated 4T1 cells for 24 h, exhibited remarkable increased phosphorylation levels of STING and interferon regulate factor 3 (IRF‐3) (Figure 4f,g). And much higher levels of IFN‐*β* were successfully induced in Raw264.7 cells than other treatments when incubated with Mito‐FFa‐treated 4T1 cells (Figure 4h). In addition, we also observed that more ATP, another ICD marker for immune activation, was triggered to release into extracellular spaces upon Mito‐FFa treatment (Figure 4i). Because IFN‐*β* and ATP are an efficient proinflammatory mediator that can recruit and activate DCs, we then fed Mito‐FFa‐treated 4T1 cells to immature BMDCs to evaluate their underlying adjuvant effects. As show in Figure 4j,k, after coculture for 24 h, much more matured BMDCs (CD80^+^/CD86^+^ in CD11c^+^) were observed in the Mito‐FFa group. Therefore, these results revealed that Mito‐FFa could induce Ox‐mtDNA leakage and promote bystander immune cells to secrete IFN‐I for maturation of DCs.

### 2.4 Enhanced Immunogenicity of Mito‐FFa‐Treated Tumor Cells

**Figure 5:**
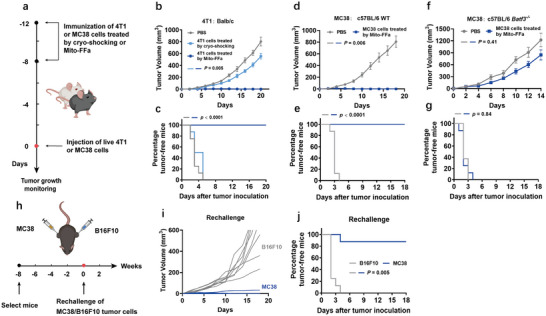
Enhanced immunogenicity of Mito‐FFa‐treated tumor cells. a) Schematic illustration of prophylactic tumor vaccination. b,c) The growth curves b) and Kaplan–Meier tumor‐free curves c) of Balb/c mice vaccinated by Mito‐FFa treated 4T1 tumor cells. Data are shown as mean ± SD (*n* = 8, one‐way ANOVA). d,e) The growth curves d) and Kaplan–Meier tumor‐free curves e) of WT c57BL/6 mice vaccinated by Mito‐FFa treated MC38 tumor cells. Data are shown as mean ± SD (*n* = 8, one‐way ANOVA). f,g) The growth curves f) and Kaplan–Meier tumor‐free curves g) of Batf3^−/−^ c57BL/6 mice vaccinated by Mito‐FFa treated MC38 tumor cells. Data are shown as mean ± SD (*n* = 8, one‐way ANOVA). h) Schematic illustration of rechallenge of MC38 and B16F10 tumor cells in the WT c57BL/6 mice vaccinated with Mito‐FFa‐treated MC38 tumor cells. i) Tumor growth curves of individual mice (*n* = 8). j) Kaplan–Meier tumor‐free curves from both MC38 and B16F10 tumors. N.S. represents nonsignificance, and *p < 0.05; **p < 0.01; ***p < 0.001.

Prior to evaluating in situ vaccine effect of Mito‐FFa, we first assessed whether the immunogenicity of tumor cells upon Mito‐FFa treatment was enhanced and their ability in eliciting antitumor immunity in vivo. Then, prophylactic vaccine experiments were performed on several tumor models. First, Balb/c mice were vaccinated subcutaneously with Mito‐FFa‐treated 4T1 tumor cells on days −12 and −8, respectively. PBS and inactivated 4T1 tumor cells (prepared by cryo‐shocking live cells in liquid nitrogen to eliminate their tumorigenicity while preserving their major structures)^[^ ^23^ ^]^ were also injected as control. On Day 0, vaccinated mice were challenged with live 4T1 tumor cells in the contralateral flank (**Figure** **5**a). As shown in Figure 5b,c, immunization of Mito‐FFa‐treated 4T1 cells resulted in 100% tumor‐free survival, which exhibited significant differences from the two control groups (both with 0% tumor‐free survival).

We then evaluated prophylactic effect in WT or Batf3^−/−^ C57BL/6 mice. Batf3 was an essential transcription factor for differentiation of cDCs, which were generally considered as the professional cross‐presenting DCs in vivo. Therefore, Batf3^−/−^ mice were defective in cross‐presentation of tumor cell antigens. Similar with 4T1 cells, Mito‐FFa‐mediated MC38 tumor cell vaccination also caused 100% tumor‐free survival in WT C57BL/6 mice (Figure 5d,e). However, the Mito‐FFa‐mediated immune activation was completely abrogated in Batf3^−/−^ C57BL/6 mice (Figure 5f,g). These results indicated that Mito‐FFa‐treated tumor cells could activate robust antitumor immunity through cross‐presentation in vivo.

To assess whether these immune responses were long‐term and tumor antigen‐specific, the survived tumor‐free WT C57BL/6 mice vaccinated by Mito‐FFa treated MC38 tumor cells were rechallenged with live MC38 and B16F10 tumor cells on both sides of mouse 2 months after vaccination (Figure 5h). The results showed that 87.5% (7/8) mice successfully rejected MC38 cells again, whereas B16F10 tumor cells grew well in all mice (Figure 5i,j). Overall, all these data suggested that Mito‐FFa‐treated tumor cells could effectively induce the generation of tumor antigen‐specific and durable immune response via cross‐presentation of DCs, which portended the great potential of Mito‐FFa for in situ tumor vaccination.

### 2.5 Intratumoral Mito‐FFa Administration Induced CD8^+^ T Cell Responses

**Figure 6:**
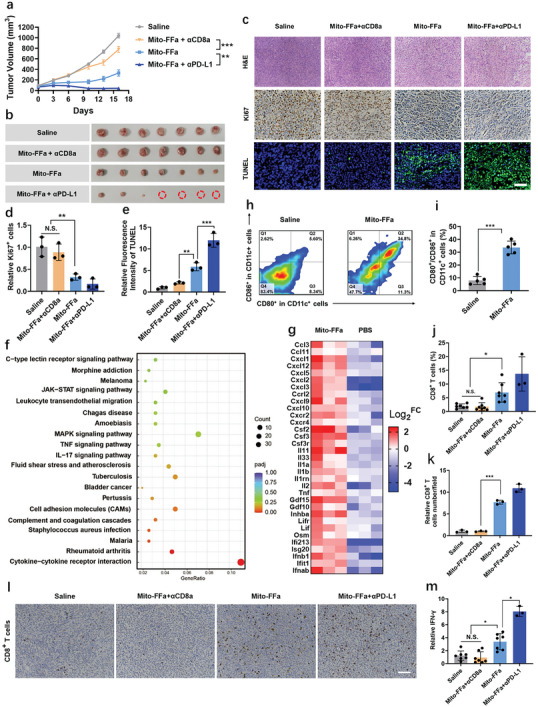
Mito‐FFa induced in situ vaccination. a) Primary tumor growth curves of 4T1 tumors after administration of Saline, Mito‐FFa, Mito‐FFa + *α*PD‐L1, and Mito‐FFa + *α*CD8a. [Mito‐FFa] = 60 µg/mouse. Both antibodies were treated via intraperitoneal injection. Data are shown as mean ± SD (*n* = 7, one‐way ANOVA). b) Images of tumor tissues collected on day 16. c) Immunofluorescence images of tumor slices stained with Ki67, TUNEL assay kit, and H&E, respectively. Scale bar = 40 µm. d,e) Quantification of Ki67 d) and TUNEL e) mean fluorescence intensity based on c). Data are shown as mean ± SD (*n* = 3, one‐way ANOVA). f) KEGG pathway enrichment analysis of differentially expressed genes in Mito‐FFa‐treated 4T1 tumors. g) Heat map depicting relative transcript levels of differentially expressed genes in Mito‐FFa or PBS‐treated 4T1 tumors (*n* = 3). h) Representative flow cytometry images of mature DCs (CD80^+^/CD86^+^ gated in CD11c^+^) in tumor‐draining lymph nodes. [Mito‐FFa] = 60 µg/mouse. i) The proportion of mature DCs in tumor‐draining lymph nodes after various treatments. Data are shown as mean ± SD (*n* = 5, one‐way ANOVA). j) The percentages of tumor‐infiltrated CD8^+^ T cells detected by flow cytometry. Data are shown as mean ± SD (*n* = 3 for Mito‐FFa + *α*PD‐L1 group and *n* = 7 for other groups, one‐way ANOVA). k,l) quantification k) and representative images l) of the immune histochemical staining of infiltrated CD8^+^ T cells. Scale bar = 100 µm. Data are shown as mean ± SD (*n* = 3, one‐way ANOVA). m) IFN‐*γ* levels within tumor tissues from different groups. Data are shown as mean ± SD (*n* = 3 for Mito‐FFa + *α*PD‐L1 group and *n* = 7 for other groups, one‐way ANOVA). N.S. represents nonsignificance, and *p < 0.05; **p < 0.01; ***p < 0.001.

Subsequently, we assessed the in situ vaccination of Mito‐FFa in poorly immunogenic 4T1 tumors. When the tumor volume reached about 100 mm^3^, Mito‐FFa was intratumorally injected with a single dose (20, 60, and 200 µg per mouse). As shown in **Figure** **6**a,b; and Figures S9–S11 (Supporting Information), 20, 60, and 200 µg per mice of Mito‐FFa treatment significantly delayed the growth of established 4T1 tumors with 47.6%, 68.1%, and 100% TGI (tumor growth inhibition), respectively. To assess the immune priming, a mid‐dose (60 µg per mouse) was selected for follow‐up evaluation. At first, immunohistochemical analysis of Ki67 revealed that the proliferation of 4T1 tumor cells was remarkably weakened by Mito‐FFa treatment (60 µg per mouse, Figure 6c,d). Meanwhile, Mito‐FFa treatment resulted in massive necrosis and apoptosis of tumor cells (Figure 6c,e). These data supported the antitumor effect of Mito‐FFa. Additionally, during the treatment, no significant changes in body weights were observed (Figure S12, Supporting Information). Even at dose of 1600 µg per injection, histological analysis of major organs and serum biochemistry (Figure S13, Supporting Information) of mice still stayed healthy, indicating the great bio‐safety of local administration of Mito‐FFa.

Next, to evaluate whether Mito‐FFa‐treated ([Mito‐FFa] = 60 µg per mouse) tumors showed immunoactive phenotype, we performed RNA sequencing (RNA‐seq) to analyze the gene expression profiles of 4T1 tumors treated by Mito‐FFa. KEGG pathway enrichment analysis showed that the most dominant pathway enriched by Mito‐Ffa treatment was cytokine–cytokine receptor interaction (Figure 6f), which would activate multiple downstream immune related signaling pathways. In which, proinflammatory cytokines, interferon stimulated genes (ifnb1, etc.,), leukocyte‐recruiting chemokines (Cxcl9, Cxcl10, etc.,) were significantly up‐regulated. RT‐qPCR and ELISA assays further validated these results (Figure S14, Supporting Information). These results indicating that the single i.t. Mito‐Ffa administration established a proinflamed microenvironment for immune priming. Encouraged by these data, we collected tumor draining lymph nodes (TDLNs) to detect DCs maturation. Compared with PBS group, Mito‐Ffa treatment significantly induced DC maturation with increased CD80^+^/86^+^ expression (Figure 6h,i). Because the up‐expression of ISGs, Cxcl9, Cxcl10 gene, and DCs activation were closely related to CD8^+^ T cells priming and recruitment, we then evaluated CD8^+^ T cells responses within tumors. Both flow cytometry and IHC analysis confirmed that abundant CD8^+^ T cells were recruited into Mito‐FFa‐treated tumors (Figure 6j‐l; and Figure S15, Supporting Information) and resulted in significantly higher levels of IFN‐*γ* (Figure 6m). Since Mito‐FFa‐mediated immunity had been proven to be cross‐presentation dependent (Figure 5), we supposed that the infiltrated CD8^+^ T cells participated in the antitumor effect of Mito‐FFa. We immunodepleted CD8^+^ T cells of mice by anti‐CD8a antibody (*α*CD8a), and observed that the therapeutic effect of Mito‐FFa was largely erased (TGI 68.7% vs 25.1%), which revealed that Mito‐FFa inhibited primary tumors growth via cross‐primed CD8^+^ T cells (Figure 6).

Successful ICB therapies relied on robust CD8^+^ T cells responses,^[^ ^24^ ^]^ so we then investigated the synergistic effect of Mito‐FFa and PD‐L1 blockade therapy. Impressively, a great synergy was observed, accounting for 96.1% TGI with 57% (4/7) complete response (Figure 6a–e). Furthermore, Mito‐FFa‐induced CD8^+^ T cell infiltration and IFN‐*γ* secretion were also obviously potentiated by *α*PD‐L1 treatment (Figure 6j–m). Therefore, all these results demonstrated that as a potent ICD inducer, Mito‐FFa could effectively render “cold tumors” into hot ones and elicit CD8^+^ T cells dependent immunity to potentiate PD‐L1 blockade‐mediated primary tumor inhibition.

### 2.6 Mito‐FFa Synergized with *α*PD‐L1 to Overcome Tumor Metastasis

**Figure 7:**
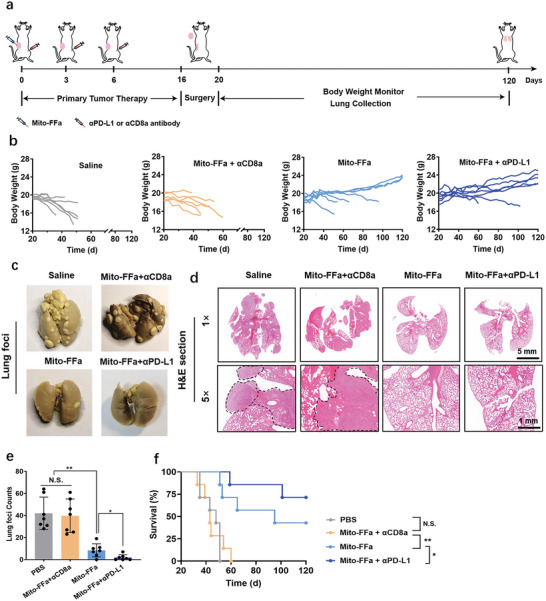
The antimetastasis effect of Mito‐FFa. a) Schematic illustration of tumor therapeutic profile. b) Body weights of individual 4T1‐tumor‐bearing mice during the treatments (*n* = 7). c,d) Images of lungs fixed by Bouin's solution c) and their H&E sections d). Metastatic areas are depicted with dashed lines. Scale bar = 5 and 1 mm. (e) Quantification of lung foci in different groups. Data are shown as mean ± SD (*n* = 7, one‐way ANOVA). f) Survival curves for mice bearing 4T1 breast tumors. N.S. represents nonsignificance, and *p < 0.05; **p < 0.01; and ***p < 0.001.

We next assessed the ability of Mito‐FFa‐mediated immune responses in controlling the formation of metastases after primary tumour remove. We excised 4T1 primary tumors and monitored the spontaneous metastasis over a long period (**Figure** **7**a). Because of the increasing metastatic burden, the body weights of mice in the Saline group decreased gradually (Figure 7b) and all mice died before day 52 (Figure 7f). However, the lifetime of mice in the Mito‐FFa group were prolonged significantly and 3/7 (42.8%) mice exhibited stable or increasing body weights, surviving until day 120 (Figure 7b,f). Meanwhile, a mass of metastatic nodules was observed in the lungs of mice treated with Saline, whereas only sporadic foci observed in the Mito‐FFa group (Figure 7c–e). However, the antimetastatic activities of Mito‐FFa were eliminated when CD8^+^ T cells were immunodepleted, exhibiting a comparable survival curve to PBS treatment (Figure 7b–f). On the other hand, in this model, Mito‐FFa exhibited excellent synergy with aPD‐L1, resulting in 71.4% long‐term survival (5/7) (Figure 7b,f) and almost cleared metastatic foci on the lungs (Figure 7c–e). We also collected spleens from the treated mice to analyze the proportion of effector memory CD8^+^ T cells (CD8^+^ Tem). The observed increase in CD8^+^ Tem after treatment with Mito‐FFa in the spleens confirmed the establishment of antitumor immune memory (Figure S16, Supporting Information). These results showed that Mito‐FFa‐induced CD8^+^ T cells response via in situ tumour vaccination was durable and could induce systemic inhibition of highly immunosuppressive solid tumors, especially when combined with ICB therapy.

## 3 Discussion

I.t. immunotherapy is a highly active area of investigation resulting in many agents, for example, immune receptor agonists, nononcolytic and oncolytic viral therapies, being tested in preclinical and clinical studies.^[^ ^25^ ^]^ I.t. delivery of immunomodulators could enhance drug dose within the lesions while effectively diminishing their systemic toxicities.^[^ ^26^ ^]^ Although advances in interventional radiology allow most tumors to be accessible for i.t. administration now, multiple drug injections are usually required to maintain therapeutic levels. In this work, we showed that a single i.t. injection of the Mito‐FFa could in situ evoke systemic antitumor immunity and potentiate anti‐PD‐L1 efficiency by coinducing CRT exposure and IFN‐I secretion. Notably, neither FF nor FFa (Figure S17, Supporting Information) were able to replicate these effects, highlighting the significant impact of mitochondrial targeting of FF/FFa in the development of tumor immunotherapy. CD8^+^ T cell responses were curial to the magnitude and duration of vaccine‐induced antitumor immunity. Mito‐FFa‐induced substantial CRT exposure ensured that APCs acquired more tumor antigens. Inducing host production of IFN‐I contributes to immunity activation in many ways,^[^ ^27^ ^]^ and we here demonstrated that IFN‐I promoted the in situ vaccine effect of Mito‐FFa by promoting cross‐presentation of antigens, DCs maturation and forming of immunoactive TME. All these events were favorable for generation of CD8 T cell‐mediated immune response.

Oxidative stress and its relevance to tumor immunotherapy are currently under extensive investigation.^[^ ^28^ ^]^ Mitochondrial complex I inhibitors are showed to be antitumor, however, previous studies mainly focused on their roles in bioenergetic exhaust and hypoxia relieving.^[^ ^29^ ^]^ Recently, immunomodulatory effects of complex I inhibitors were explored. A study in 2021 reported complex I inhibitor could suppress tumor growth by remodeling immunosuppressive TME.^[^ ^30^ ^]^ Our work reported a new application of complex I inhibition in priming antitumor immunity. Mitochondria is originated from an ancient *α*‐proteobacterium and as a “foreigner,” some constituents of mitochondria, especially mtDNA, are immunogenic and deemed as Mito‐DAMPs. Therefore, mitochondrial control of immune priming is currently of intense interest.

We were surprised at the ability of Mito‐FFa in exposing CRT. We noted that the efficiency of previously reported ICD inducers were all comparable to OxPt, however, Mito‐FFa was 35.7‐fold greater than OxPt (84.9% vs 2.38%). The reasons might include following two aspects. The first is the location of ROS generation. CRT exposure is an adaptive response of ER stress. The short diffusion distance (< 20 nm) and lifetime of ROS restrict their sphere of influence,^[^ ^31^ ^]^ however, due to the physical connections (about 10 nm) between the ER and mitochondria, mtROS produced by Mito‐FFa will readily diffuse to ER and impair their structures or functions. The second may be related to the species of the produced ROS. In our study, the ROS produced are mainly from mitochondria. MtROS mainly consisted of O_2_^−^ and H_2_O_2_ and their oxidizabilities are weaker than that of ^1^O_2_ or •OH, which are produced by other tumor therapies such as Photodynamics Therapies and Fenton Reactions, respectively.^[^ ^32^ ^]^ It is likely that increased mtROS can allow tumor cells to undergo oxidative stress and expose CRT over a sufficient period of time instead of dying rapidly.

We are also optimistic about the clinical value of Mito‐FFa. In addition to the excellent therapeutic effect, its bio‐compatibility was also laudable (Figure S10, Supporting Information). On the one hand, it should be due to the FDA approval of FFa and fact that TPP has been used to develop health‐care products (e.g., MitoQ). On the other hand, unusual metabolism patterns of Mito‐FFa may be another reason. We found that even i.t. administrated with Mito‐FFa with 1600 µg per injection, 8‐times of 200 µg per injection that could eliminate all established tumors, there was only very low amounts could be detected in the blood of mice (data not shown). This excellent i.t. retention of Mito‐FFa potentially ensures its great bio‐safety after i.t. administration. One possible explanation for this property is that cationic TPP of Mito‐FFa enables it to enter cells more rapidly (data not shown) and then be trapped within mitochondria rather than enter the bloodstream. Besides, our results also showed that Mito‐FFa‐treated tumor cells are highly immunogenic and nontumorigenic (validated in vaccinations of more than 100 mice). It is therefore plausible to believe that tumor cells pretreated with Mito‐FFa could be designed as an easily‐phagocytosed and self‐adjuvating (Ox‐mtDNA) whole‐cell vaccine for the control of established tumors.

In summary, we developed a highly potent ICD inducer by modifying mitochondria‐targeted TPP to FFa. Due to more efficient complex I inhibition, Mito‐FFa produced significantly more mtROS to induce ER stress and Ox‐mtDNA leakage, which further allowed significant CRT exposure and IFN‐I secretion for phagocytosis and cross‐priming of CD8^+^ T cells. A single i.t. Mito‐FFa injection leveraged these effects to achieve powerful in situ vaccination and elicited systemic CD8^+^ T cells responses against metastatic tumors. These results suggested that Mito‐FFa could be a promising immunostimulatory agent to improve clinical outcomes of cancer immunotherapy.

## 4 Experimental Section

### Materials

Fenofibrate, Fenofibric acid, and (4‐bromobutyl)triphenyl‐phosphonium bromide were purchased from Energy Chemical (China). Ammonia methanol solution were purchased from J&K scientific (Shanghai, China). 4′,6‐diamidino‐2‐phenylindole (DAPI) were obtained from Sigma‐Aldrich (USA). Cell mitochondrial isolation kit, ATP Assay Kit, and cell plasma membrane staining kit with DID (C1995S) were purchased from Beyotime Biotech Inc. (China). Mitotracker Red CM‐H2XRos, MitoSOX Red Mitochondrial superxide indicator, PI staining solution, and Carboxyfluorescein Diacetate Succinimidyl Ester (CFSE) were supplied by Yeasen Biotech (China). Counting Kit‐8 (CCK‐8) was obtained from Dojindo Laboratories (Japan). Recombinant mouse GM‐CSF protein and Recombinant mouse IL‐4 protein were obtained from Abcam (USA). IRF‐3 antibody (D83B9, CST), p‐IRF‐3 antibody (D6O1M, CST), STING antibody (D1V5L, CST), p‐STING (D8F4W, CST), *β*‐actin antibody (13E5, CST), GADPH antibody (Abcam, USA), HSP‐60 (Abcam, USA), and Histon‐H3 (Abcam, USA), CHOP Polyclonal Antibody (YT0912, ImmunoWay), Phospho‐eIF2*α* antibody (D9G8, CST), eIF2*α* antibody (D7D3, CST), FITC‐8‐hydroxyguanine antibody (bs‐1278R‐FITC, Bioss). Mouse IFN‐*γ*‐precoated enzyme‐linked immunosorbent assay (ELISA) Kit was obtained from DAKEWEI (China). Anti‐Calreticulin‐ER Marker (Alexa Fluor 488), TUNEL Assay Kit – BrdU‐Red, and Anti‐Ki67 antibody were supplied by Abcam (USA). PE antimouse CD3 antibody, APC antimouse CD8a antibody, PE antimouse CD80 antibody, Alexa Fluor 647 antimouse CD86 antibody, Alexa Fluor 488 antimouse CD11c antibody, and Ultra‐LEAF Purified antimouse CD8a (clone: 53–6.7) were purchased from BioLegend (USA). In vivo MAb antimouse PD‐L1(B7‐H1) (clone: 10F.9G2) was purchased from BioXcell (USA). The mouse CT26 colorectal cancer cells and 4T1 breast cancer cells were purchased from China Type Culture Collection, supplied by the American Type Culture Collection. BALB/c and c57BL/6 mice were obtained from the Yangzhou University Medical Centre (China).

### Synthesis and Characterization Data of 4‐Aminobutyltriphenylphosphonium Bromide and Mito‐FFa

4‐Aminobutyltriphenylphosphonium bromide (TPP‐Amine) was prepared as follows. Briefly, a solution of (4‐bromobutyl)triphenyl‐phosphonium bromide (1 g, 2.512 mmol) in ammonia methanol solution (7N, 100 mL, 700 mmol) was incubated under room temperature in a sealed flask for 3 days. After evaporation of the solvent the residue was viscous liquid. After vacuum drying, a colorless foam was obtained and was used without further purification.

Fenofibric acid (160 mg, 0.5 mmol) was dissolved in dichloromethane (20 mL) containing Triethylamine (160 µL, 1.5 mmol). TPP‐Amine 1 (369 mg, 0.75 mmol), 1‐Hydroxybenzotriazole (Hobt, 81 mg, 0.60 mmol), EDCI (135 mg, 0.7 mmol) were added to the solution in sequence and the mixture was incubated under room temperature for 12 h. Then, saturated NaCl solutions were used to wash the mixture for three times and Mito‐FFa was obtained using rotary evaporation. Yield: 91%. MS: C_39_H_38_ClNO_3_P^+^: Cal. 634.23, found 634 (M^+^).^1^H NMR (400 MHz, DMSO‐d6) *δ* 8.23–8.20 (m, 1H), 7.91–7.86 (m, 3H), 7.80–7.72 (m, 11H), 7.69–7.60 (m, 7H), 6.93–6.89 (m, 2H), 3.60–3.53 (m, 2H), 3.17–3.12 (m, 2H), 1.64–1.60 (m, 2H), 1.46–1.44 (m, 8H). ^13^C NMR (150 MHz, DMSO‐d6) *δ* 193.2, 133.5, 131.2, 130.3, 130.2, 129.6, 128.6, 118.8, 118.2, 118.2, 80.6, 37.6, 29.5, 29.4, 25.0, 19.9 (d, *J =* 49.8), 19.2 (d, *J* = 4.2). Although solvent peaks of ethyl acetate and dichloromethane appeared in the ^13^C NMR spectrum, its presence did not interfere with the identification of Mito‐FFa. Mito‐FFa powder was collected for elemental analysis by X‐ray photoelectron spectroscopy (XPS, Thermo Scientific K‐Alpha).

### Cellular Uptake and Mitochondrial Accumulation of FF and Mito‐FFa

4T1 tumor cells were grown in 100 mm dishes to about 5 × 10^6^ per dish and then treated with 8 µm FF and Mito‐FFa. After 12 h incubation, the culture supernatant was collected. On the other hand, mitochondria were isolated using the cell mitochondrial isolation kit (Beyotime) according to the manufacturer's recommended protocol and lysed by an ultrasonic crusher. Then, threefold volume of acetonitrile was added to the solution to precipitate proteins in medium and mitochondrial lysate. The supernatant was collected by centrifugation (12 000 rpm, 10 min) for high‐performance liquid chromatography (HPLC) analysis. Analyses were performed on a InertsilODS‐3 V (150 × 4.6 mm × 5 µm) column (GL Sciences Inc.). Eluent A (0.1% trifluoroacetic acid in H_2_O), eluent B (acetonitrile). Gradient: T/%B:0/5, 20/90, 27/5, 30/5, Diluent: Water: Acetonitrile = 1:1, Flow rate: 1.0 mL min^−1^. The wavelength for the detection of Mito‐FFa was 284 nm.

### Cytotoxicity Evaluation

To investigate the cytotoxicity of FF ([FF] = 10, 20, 40, 80, 100, 140, 180, 220 µm), FFa ([FFa] = 10, 20, 40, 80, 100, 140, 180, 220 µm), and Mito‐FFa ([Mito‐FFa] = 0, 1, 2.5, 5, 10, 20, 40, 80 m), 4T1 cells, MDA‐MB‐231, CT26, AML12 cells were seeded into 96‐well plates with a density of 6 × 10^3^ per well. After attachment, the cells were incubated with various drugs for 24 h. Then, all treatments were removed and cells were washed by PBS. Finally, the tumor cell viability was determined by CCK‐8 assay (Dojindo, Japan) and ic50 curves were plotted by GraphPad Prism 8.0.

### Mitochondrial Respiratory Complex I Inhibition of Mito‐FFa and FF

4T1 tumor cells were grown in 100 mm dishes to about 5 × 10^6^ per dish. Then, Mito‐FFa and FF [8 µm] were added and incubated for 6 h. Then, cells were collected to detect the activity of complex I using Mitochondrial Respiratory Complex I Activity Assay Kit (Boxbio, China) according to the manufacturer's protocol.

### Mitochondrial ROS Detection

4T1 cells were seeded into 96‐well plates with a density of 6 × 10^3^ per well. After attachment, Mito‐FFa (8 µm), FFa (123 µm), and FF (80 µm) were added and incubated for 6 h. Then, all treatments were removed and cells were coincubated with the fresh dulbecco's modified eagle medium (DMEM) containing Mitotracker Red CM‐H2XRos (Invitrogen, US) for 30 min at 37 °C in the dark. After that, DAPI was then used to stain nucleus. Then, tumor cells were observed by Nikon Eclipse Ti (Japan). For mitochondrial superoxide anion detection, after various treatments above, 5 × 10^5^ tumor cells in suspension were incubated with MitoSOX Red Mitochondrial superxide indicator (Yeasen Biotech, China) for 30 min at 37 °C in the dark. Then, cells were washed and analyzed by flow cytometry (BD Calibur).

### Mitochondrial Potential Detection

4T1 tumor cells were seeded into confocal dishes with a density of 2 × 10^5^ per well. After attachment, cells were co‐incubated with CCCP (10 µm) for 2 h, PBS, Mito‐FFa (8 µm), and FF (80 µm) for 6 h, respectively. Then, all treatments were removed and the mitochondrial inner transmembrane potential (ΔΨm) was evaluated using Mitochondrial Membrane Potential Assay Kit with JC‐1 (Solarbio, China). Meanwhile, 4T1 cells after various treatments were harvested by trypsinization and analyzed by flow cytometry (BD Calibur) after JC‐1 staining.

### Subcellular Structures Observation by TEM

4T1 cells were grown in 100 mm dishes to about. Then, cells were treated with PBS and Mito‐FFa [8 µm] for 6 h. Next, cells were collected and 1 mL of 2.5% w/w glutaraldehyde in PBS solution was added to the cell pellets and stored at 4 °C overnight. Afterward, cells were fixed with 1% w/w OsO4 in PBS for 1 h, dehydrated with ethanol, embedded in resin, cut into ultrathin sections, placed on the copper grids. Finally, subcellular structures were observed under the transmission electron microscopy (HITACHIHT7800) and ER tubule width was calculated by Image J.

### CRT Exposure Analysis

4T1 tumor cells were seeded into confocal dishes with a density of 2 × 10^5^ per well. After attachment, cells were coincubated with PBS, OxPt (40 µm) Mito‐FFa (8 µm), FFa (123 µm), and FF (80 µm) at their respective IC_50_ concentrations for 6 h. Then, all treatments were removed. After washed with PBS 3 times, cells were incubated with Alexa Fluor488‐CRT antibody (diluted 1:500 with 3% BSA, ab196158, Abcam) at 4 °C for 1 h. After washed by PBS twice, nucleus was further stained with DAPI. The immunofluorescence images were obtained via Olympus FV3000 LSCM and analyzed with Image J. For flow cytometry analysis, treated cells were harvested by trypsinization and stained with Alexa Fluor 488‐CRT antibody at 4 °C for 1 h. Next, after washed by PBS twice, PI (Yeasen Biotech, China) was added to stain and exclude dead cells. Finally, tumor cell suspensions were detected using flow cytometry (BD Calibur).

### BMDCs Differentiation

To prepare BMDCs, bone marrow was collected from the femurs of female murine WT C57BL/6 using a needle to flush the marrow from the bone. Red blood cells were lysed with ACK lysis buffer (Life Technologies) as described by the vendor. Then, the obtained cells were cultured in 100 mm plates for 8–10 d in RPMI 1640 medium supplemented with 10% FBS, 100 mm *β*‐mercaptoethanol, penicillin/streptomycin, 10 ng mL^−1^ recombinant murine IL‐4 (Abcam, USA) and 20 ng mL^−1^ GM‐CSF (Abcam, USA). Half of the media was replaced every 2–3 days with fresh media and cytokines. Before use, FITC‐anti‐CD11c antibody was used to stain cells to confirm the successful differentiation of BMDCs by flow cytometry (BD Calibur).

### Phagocytosis Assay and Activation of BMDCs In Vitro

4T1 tumor cells were treated by PBS, OxPt [40 µm], Mito‐FFa [8 µm], and FF [80 µm] for 6 h, and then harvested and stained with DID (5 µm, Beyotime Biotech Inc., China) for 30 min at 37 °C. Meawhile, Raw264.7 or BMDCs were stained with CFSE (5 µm, Yeasen Biotech, China) for 30 min at 37 °C. Then, tumor cells were co‐cultured with Raw264.7 or BMDCs at a ratio of 1:1 in 6‐well plates for 24 h. Finally, all cells were harvested and analyzed by flow cytometry (BD Calibur). Phagocytosis (%) was calculated according to the formula, number of phagocytes harboring cancer cells (right‐upper quadrant)/total number of phagocytes (right quadrants). For CLSM analyses, treated 4T1 tumor cells and BMDCs upon staining were seeded into confocal dishes together. After cocultured for 24 h, phagocytosis of tumor cells by BMDCs was then observed with Olympus FV3000 LSCM. For evaluating the activation of BMDCs, 4T1 cells upon treatments of PBS, OxPt [40 µm], Mito‐FFa [8 µm], and FF [80 µm], respectively, were coincubated with immature BMDCs for 24 h. Then, cells were harvested and stained with anti‐CD11c, anti‐CD80, and anti‐CD86 at 4 °C for 1 h. After washed by PBS twice, cell suspensions were analyzed by flow cytometry (BD Calibur).

### Quantification of mtDNA in Cytosolic Extracts

4T1 cells were first treated with PBS, OxPt [40 µm], FF [80 µm], and Mito‐FFa [8 µm], respectively, for 6 h. Then cells (≈8 × 10^6^) were divided into 2 equal aliquots. One was used to extract total mtDNA using FastPure Blood/Cell/Tissue/Bacteria DNA Isolation Mini Kit (Vazyme, China) served as normalization controls. The other was resuspended in 500 µL buffer containing 150 mm NaCl, 50 mm HEPES (pH 7.4), and 20 µg mL^−1^ digitonin. The homogenate was incubated for 10 min to allow selective plasma permeabilization, and then centrifuged at 17 000 g for 10 min to pellet any remaining cellular debris, yielding cytosolic preparations without nuclear, mitochondrial, and ER contamination, which were confirmed by Western blot of GADPH, Hsp‐60, and Histon‐H3, respectively. DNA was then purified from these pure cytosolic fractions using the DNA Isolation Mini Kit mentioned above. Then, qPCR (c1000, Bio‐Rad) was performed for both whole‐cell extracts and cytosolic fractions using nuclear DNA primers (Tert) and mtDNA primers (Dloop2 and Cytb), and the Ct values obtained for mtDNA abundance for whole‐cell extracts served as normalization control for the mtDNA values determined from the cytosolic fractions. The primers are listed as follow

Mouse‐mtDNA CytB (F) GCTTTCCACTTCATCTTACCATTTA
Mouse‐mtDNA CytB (R) TGTTGGGTTGTTTGATCCTG
Mouse‐mtDNA Dloop2 (F) CCCTTCCCCATTTGGTCT
Mouse‐mtDNA Dloop2 (R) TGGTTTCACGGAGGATGG
Mouse‐nucDNA Tert (F) CTAGCTCATGTGTCAAGACCCTCTT
Mouse‐nucDNA Tert (R) GCCAGCACGTTTCTCTCGTT

### Detection of ATP Release

ATP concentrations in the supernatant of tumor cells upon the indicated treatments were measured by ATP Assay Kit (Beyotime, China), according to the manufacturer's protocol. Luminescence and absorbance were measured by microplate reader (VICTOR Nivo).

### The Fluorescence Image of Oxidized mtDNA

4T1 cells were treated by PBS, OxPt [40 µm], Mito‐FFa [8 µm], and FF [80 µm] for 6 h. The treated 4T1 cells were incubated with MitoTracker Red CMXRos (Yeasen, China) at 37 °C for 45 min and cells were next fixed with cold methanol at ‐20 °C for 10 min and permeabilized with 0.1% Triton X‐100. Then, 4% paraformaldehyde was used to refix cells at RT for 10 min, and NaOH solution ([50 mm] in 50% ethanol) was added to denature DNA. After washed by PBS three times, cells were blocked with 1% BSA/PBS, and incubated with an FITC‐8‐hydroxyguanine antibody (bs‐1278R‐FITC, Bioss) at 4 °C for 1 h. Then, nuclei were stained with DAPI (Beyotime, China). Finally, cells were observed with Olympus FV3000 LSCM.

### Characterization of cGAS‐STING Activation and IFN‐*β* Secretion

4T1 cells were grown in 6‐well plates to 5 × 10^5^ per well. Then, cells were coincubated with PBS, OxPt (40 µm), FF (80 µm), FFa (123 µm), and Mito‐FFa (8 µm) for 6 h. Then, all treatments were removed and Raw264.7 cells were added to coincubate for another 24 h. To assay the activation of cGAS‐STING pathway, the proteins of the mixed cells were isolated using the mixture of nondenatured Tissue/Cell Lysate Kit (Solarbio) with broad spectrum protease inhibitor cocktail (ethylenediaminetetraacetic acid (EDTA) free, BOSTER) and broad‐spectrum phosphatase inhibitor cocktail (EDTA free, BOSTER). Then, 40 µg protein was used for western blotting analysis (p‐STING, STING, p‐IRF3, IRF3, GADPH). To detect the production of IFN‐*β*, treated 4T1 cells were coincubate with Raw264.7 cells for 24 h. And then, IFN‐*β* in supernatants was collected and detected by ELISA kit (Biolegend, USA).

### Prophylactic Vaccination of Mito‐FFa‐Treated Tumor Cells

To detect the vaccination effects, 1 × 10^5^ 4T1 or MC38 cells treated with Mito‐FFa were injected subcutaneously into the left flank of mice on day −12 and day −8 (4T1 cells for Balb/c mice, MC38 cells for WT c57BL/6 and *Batf3* ^−/−^c57BL/6 mice). As a control, two groups of mice were injected with PBS or inactivated tumor cells, respectively. Inactivated tumor cells were prepared by cryo‐shocking of liquid nitrogen. In brief, 4T1 cells were centrifuged at 300 g for 5 min and suspended in noncontrolled‐rate cell cryopreservation medium at a density of 1 × 10^6^ mL^−1^. The cell‐containing medium was immersed in liquid nitrogen for 12 h. Before use, the medium was thawed at 37 °C and 4T1 cells were pelleted at 300 g for 5 min. After washing with PBS, 4T1 cells were suspended in PBS for subsequent experiments. On Day 0, 1 × 10^5^ live 4T1 or MC38 cells per mouse were injected in the contralateral flank of all mice. Then, the growth of contralateral tumor was daily recorded. For rechallenge, 2 months after vaccination, 1 × 10^5^ living MC38 and B16F10 cells were subcutaneously injected in in two flanks of tumor‐free WT c57BL/6 mice, respectively and the growth of these tumors was daily recorded.

### Antitumor Capacity of Mito‐FFa

All the animals were obtained from Yangzhou University Medical Center (Yangzhou, China). All animal experiments were performed in accordance with the Guidelines for Care and Use of Laboratory Animals of Nanjing University and the experiments were approved by the Animal Ethics Committee of Nanjing University (IACUC‐D2202160).

Female BALB/c mice were subcutaneously injected with 5 × 10^5^ 4T1 tumor cells in right flank. When the tumor volume reached about 100 mm^3^, mice were divided into 8 groups including Saline, FF/FFa (0.084 µmol per mouse), Mito‐FFa (0.028 µmol, 20 µg per mouse), Mito‐FFa (0.084 µmol, 60 µg per mouse), Mito‐FFa (0.280 µmol, 200 µg per mouse), Mito‐FFa + *α*PD‐L1, and Mito‐FFa + *α*CD8a ([Mito‐FFa] = 60 µg per mouse, [*α*PD‐L1] = 10 mg kg^−1^, [*α*CD8a] = 10 mg kg^−1^). *α*PD‐L1 (BioXcell, USA) and *α*CD8a (Dakewe Biotech, China) were treated via intraperitoneal injection every 3 days upon Mito‐FFa local treatment. Tumor size and body weights of each group were recorded every 3 days. Tumor volume was calculated according to the formula: width2 × length/2. After 16 days of monitor, tumor tissues were collected by surgery for H&E, TUNEL, and ki67 staining.

For assessing the bio‐safety of Mito‐FFa treatment, PBS or 1600 µg of Mito‐FFa were intratumorally injected in five 4T1 tumor bearing mice. After 16 days, the blood and major organs of mice from both groups were collected for serum biochemistry analysis and H&E staining, respectively.

### RNA‐seq Analysis

Total RNA was extracted from the 4T1 tumor tissue 24 h (i.t. injection of Mito‐FFa,) after treatment as specified experiments using TRIZOL Reagent (Invitrogen) and genomic DNA was removed using DNase I (TaKara). Then the quality of RNA was quantified using the ND‐2000 (NanoDrop Technologies). RNA sample, satisfied with the following conditions including OD260/280 = 1.8–2.2, OD260/230 ≥ 2.0, RIN ≥ 6.5 and 28S:18S ≥ 1.0, was used to construct sequencing library. RNA‐seq transcriptome libraries were prepared following TruSeqTM RNA sample preparation Kit from Illumina (San Diego, CA), using 1 µg of total RNA. To identify DEGs (differential expression genes) between the two different samples, the expression level for each transcript was calculated using the fragments per kilobase of exon per million mapped reads (FRKM) method. R statistical package edge R (Empirical analysis of Digital Gene Expression in R, http://www.bioconductor.org) was used for differential expression analysis. The fold change was calculated by comparing against the average normalized gene expression values of saline‐treated tumors. All statistical analysis and clustering analysis were performed in R iDEP.91.

### Validation of RNA‐seq Results by ELISA and RT‐qPCR Assay

IL‐2 and TNF‐*α* within tumors on Day 5 after treatments was detected by ELISA kits (Dakewe Biotech Co., Ltd.). For RT‐qPCR, total RNA was extracted from tumors that treated by Mito‐FFa or Saline for 24 h using the RNA‐easy Isolation Reagent (Vazyme, China) and reverse transcribed using a Hifair III 1st Strand cDNA Synthesis Kit (Yeasen, China) according to the manufacturer's instructions. The qPCR was performed using a SYBR Premix Ex Taq II kit (TaKaRa, Japan) with the Roche LightCycler 96 Real‐Time PCR System (Roche, Switzerland). Expression values were normalized to GADPH and fold induction was normalized to Saline group by using 2^−ΔΔCt^ Method. The primers are listed as follow

Mouse Cxcl9 (F) CCTAGTGATAAGGAATGCACGATG
Mouse Cxcl9 (R) CTAGGCAGGTTTGATCTCCGTTC
Mouse Cxcl10 (F) AATGAGGGCCATAGGGAAGC
Mouse Cxcl10 (R) ATCGTGGCAATGATCTCAACAC
Mouse IL‐2 (F) AACCTGAAACTCCCCAGGAT
Mouse IL‐2 (R) CATCATCGAATTGGCACTCA
Mouse ISG‐20 (F) AGAGATCACGGACTACAGAA
Mouse ISG‐20 (R) TCTGTGGACGTGTCATAGAT
Mouse Ifnb1 (F) TCCAGCTCCAAGAAAGGACG
Mouse Ifnb1 (R) TTGAAGTCCGCCCTGTAGGT
Mouse Tnf (F) GTGACCTGGACTGTGGGCCTC
Mouse Tnf (R) GGCTCTGTGAGGAAGGCTGTG
Mouse GADPH (F) TGACGTGCCGCCTGGAGAAA
Mouse GADPH (R) AGTGTAGCCCAAGATGCCCTTCAG

### Evaluation of Antitumor Immune Responses

For the analysis of DC maturation, TDLNs were collected 5 days post different treatments. The collected lymph nodes were ground and filtered through 100 µm cell strainers to prepare single cell suspensions. Then, the cells were stained with Alexa Fluor 488 antimouse CD11c, Alexa Fluor 647 antimouse CD86, and PE antimouse CD80 antibodies (BioLegend, USA) and then detected by flow cytometry (BD Calibur). For detecting the infiltration of CD8^+^ T cells, tumors of mice in different groups were harvested at observe endpoint (Day 16) and cut into small pieces. Then, samples were digested in 2 mL RPMI containing 500 µg mL^−1^ collagenase IV (Invitrogen), 100 µg mL^−1^ hyaluronidase, and 20 U mL^−1^ DNase (Macklin) at 37 °C for 30 min and single cell suspensions were prepared after filtered through a 300‐mesh cell screen. Cells were next stained with PE antimouse CD3 and APC antimouse CD8a (BioLegend, USA) for flow cytometry analysis. Tumor tissues were also collected for immunohistochemical staining to further evaluate tumor infiltration. Furthermore, IFN‐*γ* within tumors was detected by ELISA kits (Dakewe Biotech Co., Ltd.).

### Analysis of Memory T Cells

Spleens were harvested from mice treated by a single i.t. injection of Saline or Mito‐FFa (60 µg per mouse) on Day 40. Use the flat end of the plunger to mince the spleen and then passed strainer. Red blood cells were removed by RBC Lysis and a single cell suspension were prepared. PE antimouse CD62L, FITC antimouse CD8a, APC antimouse CD44 antibodies (BioLegend, USA) were introduced to identify effector memory T cells (Tem, CD8^+^ CD62L^−^ CD44^+^) by flow cytometry (BD Calibur).

### Tumor Metastasis Detection

To evaluate the antimetastasis effects of Mito‐FFa, the primary tumors of all mice were carefully removed via surgery at Day 16–20, and the wounds were sutured in time to ensure the survival of the mice. Then, the body weights were daily recorded and lasted for another 100 days. Besides, lungs from mice that died or were at the trial endpoint were collected and fixed with Bouin's solution for lung metastatic foci detection.

### Statistical Analysis

A two‐tailed Student's *t*‐test was performed for the comparison of two groups. For multiple comparisons, a one‐way or two‐way analysis of variance (ANOVA) test was performed. Then, *p* value > 0.05 represented nonsignificance (N.S.), and *p* value < 0.05 represented statistically significant.

## Conflict of Interest

The authors declare no conflict of interest.

## Supporting information

Supporting Information

## Data Availability

The data that support the findings of this study are available from the corresponding author upon reasonable request.
