# Supplementary material for: In Situ Vaccination with Mitochondria‐Targeting Immunogenic Death Inducer Elicits CD8+ T Cell‐Dependent Antitumor Immunity to Boost Tumor Immunotherapy
Source: Adv Sci (Weinh). 2023 May 1;10(20):2300286. doi: 10.1002/advs.202300286 (PMC10369267; doi:10.1002/advs.202300286)
Supplement: Supplementary file 1 — Supporting Information [file ADVS-10-2300286-s001.pdf]

ADVANCED SCIENCE
Open Access

Supporting Information

for *Adv. Sci.*, DOI 10.1002/advs.202300286

In Situ Vaccination with Mitochondria-Targeting Immunogenic Death Inducer Elicits CD8$^+$ T Cell-Dependent Antitumor Immunity to Boost Tumor Immunotherapy

Yuxiang Wang, Weiran Wang, Rong Gu, Jing Chen, Qian Chen, Tingsheng Lin, Jinhui Wu, Yiqiao Hu* and Ahu Yuan**

Supporting Information

In situ Vaccination with Mitochondria-Targeting Immunogenic Death Inducer Elicits CD8$^+$ T cell-dependent Antitumor Immunity to Boost Tumor Immunotherapy.

Yuxiang Wang[1,†], Weiran Wang[1,†], Rong Gu[1], Jing Chen[1], Qian Chen[1], Tingsheng Lin[1,3,], Jinhui Wu[1,2], Yiqiao Hu[1,2,*] and Ahu Yuan[1,2,*]*

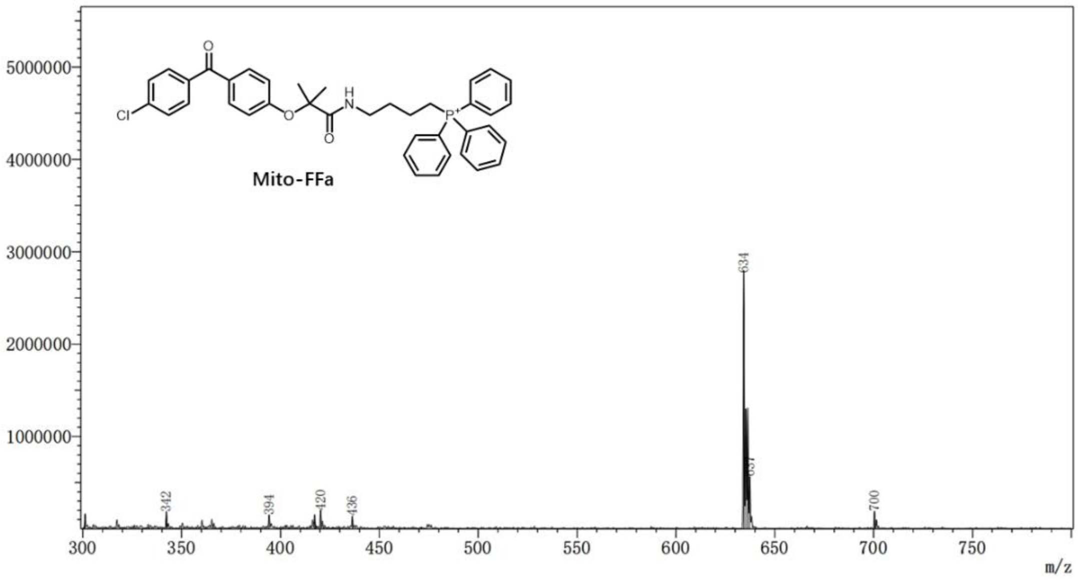

Figure S1 ESI-MS (positive mode) spectra of Mito-FFa.

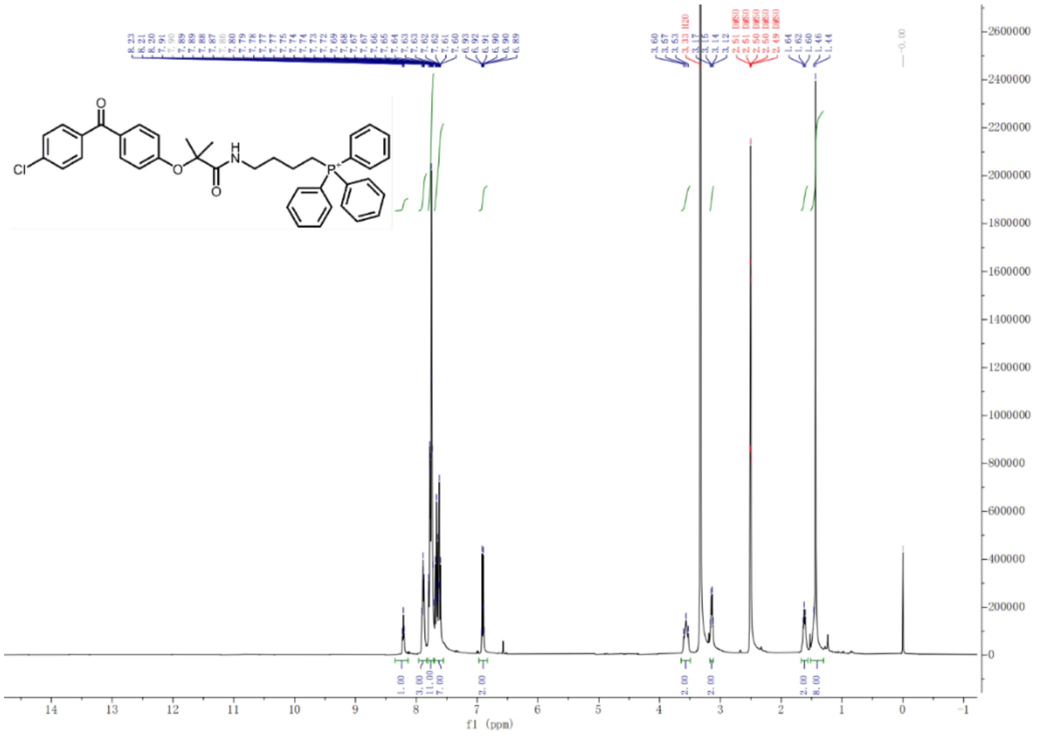

Figure S2 ^{1}H NMR (400 MHz, DMSO-d6) spectra of Mito-FFa.

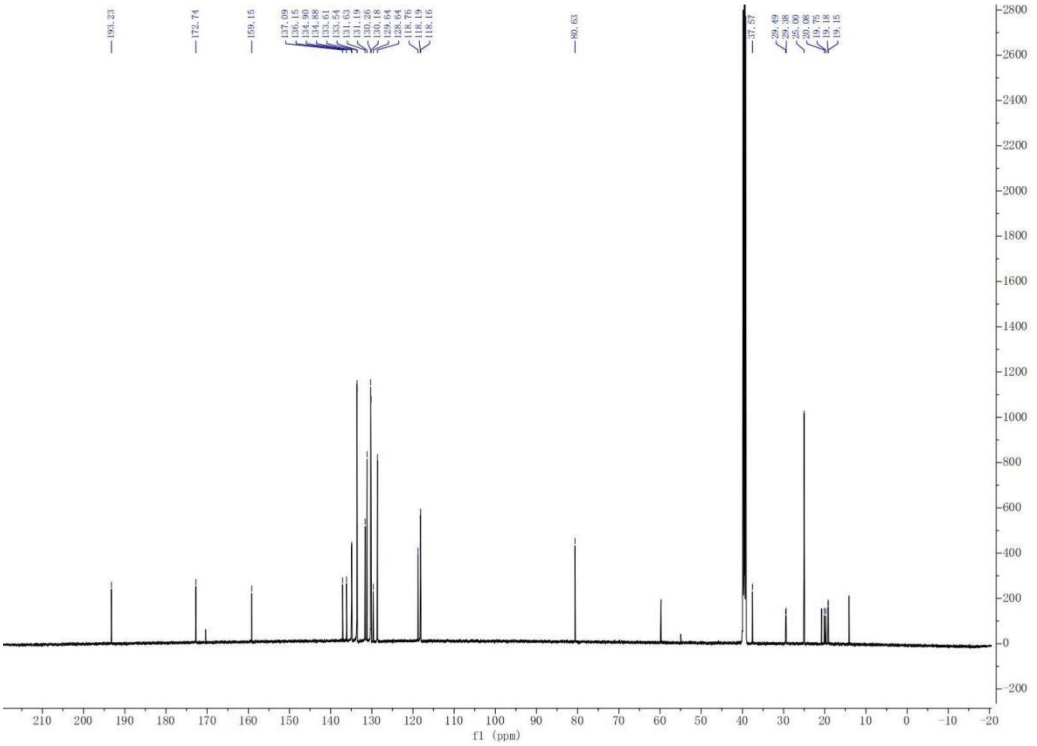

Figure S3 ^{13}C NMR (150 MHz, DMSO-d6) spectra of Mito-FFa.

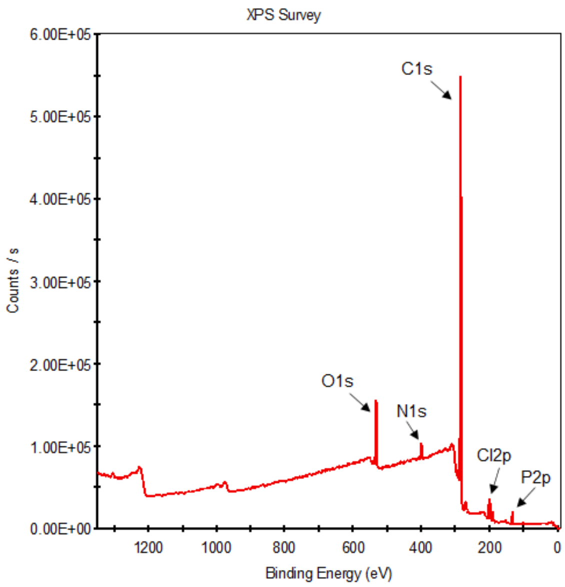

Figure S4 Elemental analysis of Mito-FFa powders detected by X-ray photoelectron spectroscopy (XPS).

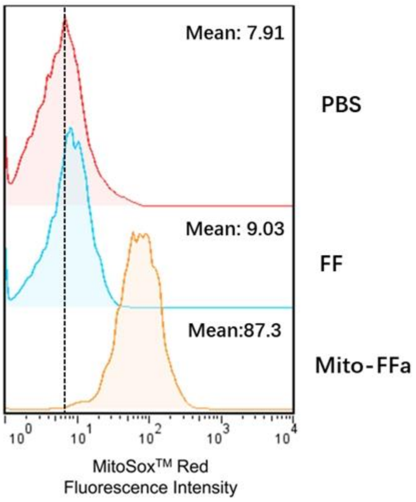

Figure S5 Representative mean fluorescence intensity (MFI) of mitochondrial superoxide production within 4T1 breast tumor cells treated with either FF (80 μM) or Mito-FFa (8 μM), respectively.

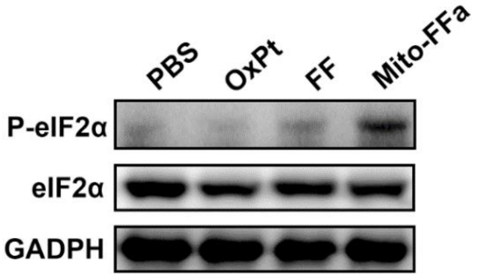

Figure S6 Western blot of P-eIF2α, eIF2α and GADPH in 4T1 tumor cells after different treatments.

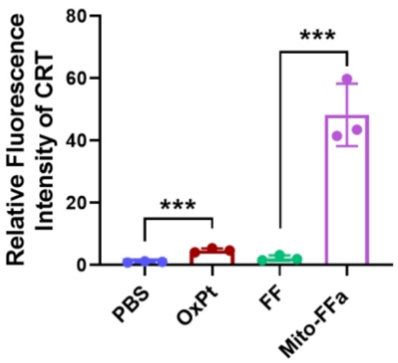

Figure S7 Quantification of relative CRT mean fluorescent intensity based on Fig. 3g. 4T1 cells were treated with OxPt (40 μM), FF (80 μM) or Mito-FFa (8 μM) for 6 hours. Data are shown as mean ± SD (n = 3, one-way ANOVA).

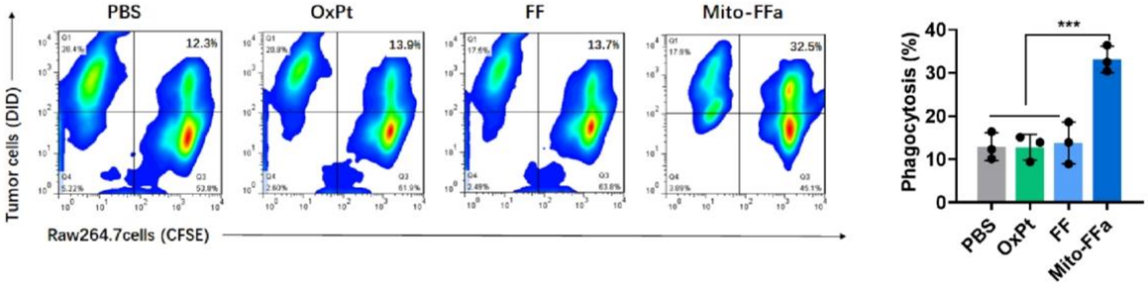

Figure S8 Representative flow cytometry plots and their quantification of Raw264.7 phagocytosing 4T1 tumor cells upon different treatments.

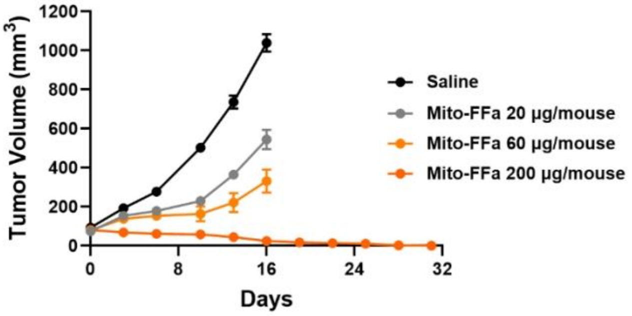

Figure S9 Primary tumor growth curves of 4T1 tumors after local administration of Saline, Mito-FFa with different dose (20 μg, 60 μg and 200 μg/mouse). Data are shown as mean ± SD (n = 7, one-way ANOVA).

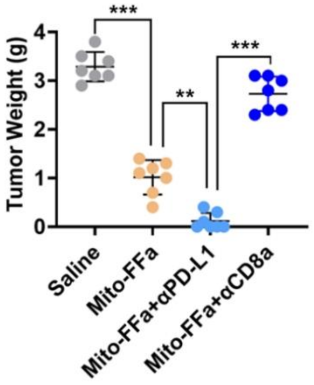

Figure S10 Tumor weights of mice in different groups. Data are shown as mean ± SD (n = 7, one-way ANOVA).

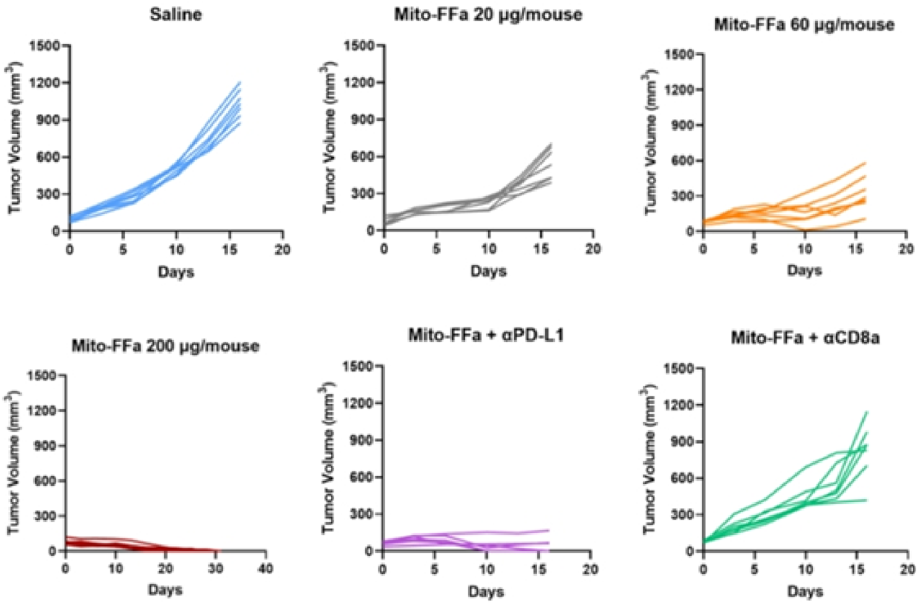

Figure S11 Growth curves of individual tumor after treatments in different groups.

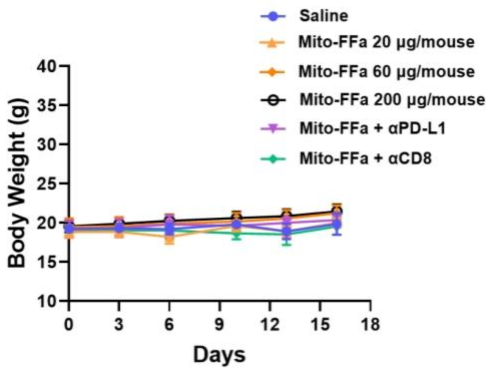

Figure S12 Body weights of mice during treatments. Data are shown as mean ± SD, n = 7.

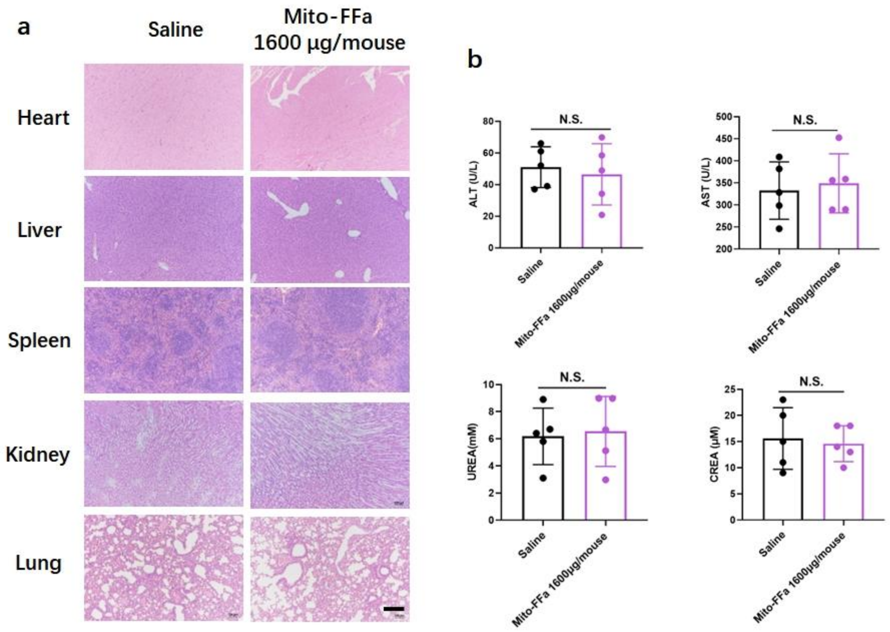

Figure S13 (a) H&E staining of major organs (heart, liver, spleen, lung, kidney) harvested on day 16 after different treatments. Scale bar = 400 μm. (b) Serum biochemistry data indicating liver and kidney function. Data are shown as mean ± SD (n = 5, one-way ANOVA).

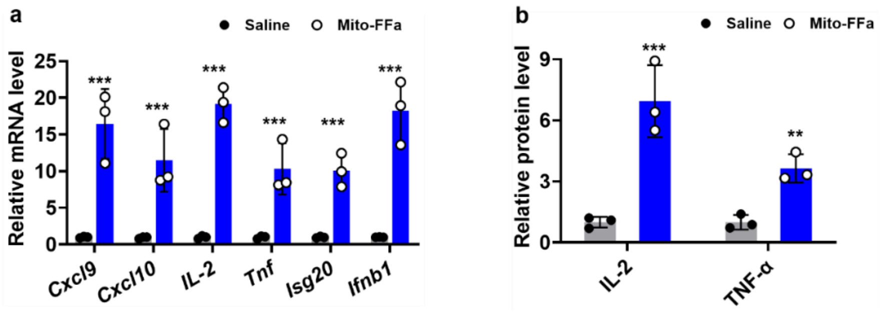

Figure S14 (a) The mRNA expression levels of Cxcl9, Cxcl10, IL-2, Tnf, Isg20, Ifnb1 within tumor tissues from different groups. (b) IL-2 and TNF-α levels within tumor tissues from different groups. Data are shown as mean ± SD (n = 3, one-way ANOVA). N.S. represents nonsignificance, and *p < 0.05; **p < 0.01; ***p < 0.001.

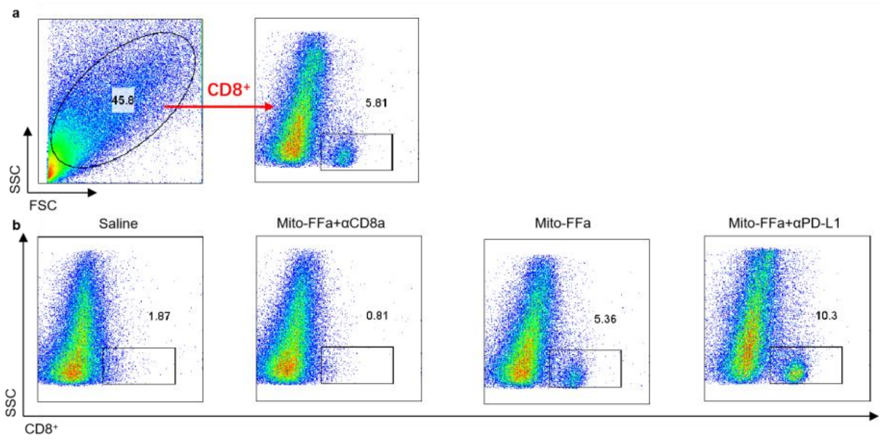

c

	S1	S2	S3	S4	S5	S6	S7
**Saline**	0.83	1.54	1.87	1.47	2.91	2.02	3.12
**Mito-FFa+αCD8a**	0.81	0.62	1.36	1.56	1.01	2.19	4.8
**Mito-FFa**	6.83	5.36	3.45	13.4	9.72	3.56	5.81
**Mito-FFa+αPD-L1**	20.9	10.3	9.85				

Figure S15 (a-b) The gating strategy (a) and representative flow cytometry images (b) of Figure 6j illustrating the population of $CD8^+$ T cells in tumors. (c) Raw data of Figure 6j.

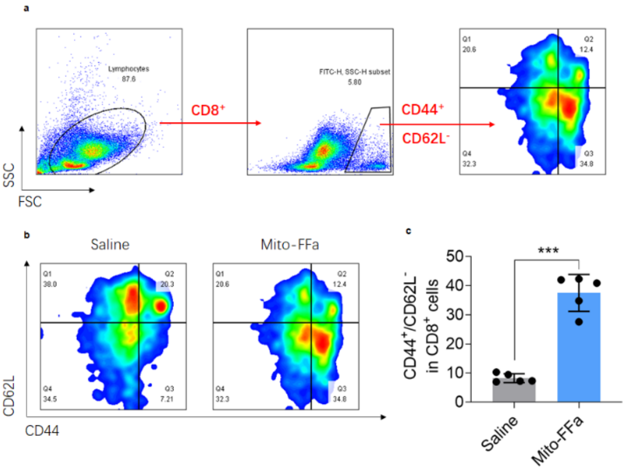

Figure S16 (a) The gating strategy illustrating the population of effector memory T cells in spleen. (b-c) Representative flow cytometry images (b) and quantification (c) of CD44^{+}/CD62L^{-} memory CD8 T cells in spleens of treated 4T1-bearing mice. Data are shown as mean ± SD (n = 5, Student's t-test). N.S. represents nonsignificance, and *p < 0.05; **p < 0.01; ***p < 0.001.

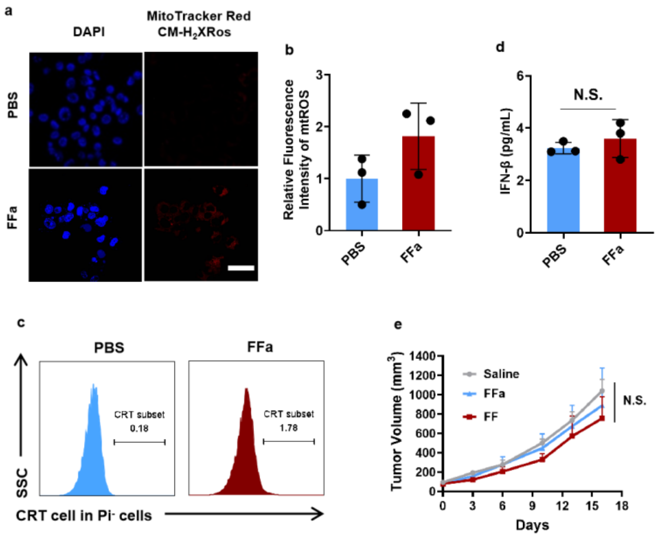

Figure S17 (a, b) The fluorescence images (a) and quantification (b) of mtROS induced by PBS and FFa. Scale bar = 50 μm. Data are shown as mean ± SD (n = 3, one-way ANOVA). (c) Representative flow cytometry images of PI^{-}/CRT^{+} 4T1 tumor cells. (d) Detection of IFN-β levels in the Raw264.7/ treated 4T1 tumor cells co-culture media by ELISA. Data are shown as mean ± SD (n = 3, one-way ANOVA). (e) Primary tumor growth curves of 4T1 tumors after a single i.t. injection of Saline, FF and FFa. Data are shown as mean ± SD (n = 7, one-way ANOVA).
